# Menopause and facial skin microbiomes: a pilot study revealing novel insights into their relationship

**DOI:** 10.3389/fragi.2024.1353082

**Published:** 2024-03-21

**Authors:** Martin Patrick Pagac, Martin Stalder, Remo Campiche

**Affiliations:** DSM-Firmenich, Perfumery and Beauty, Kaiseraugst, Switzerland

**Keywords:** menopause, aging, skin, microbiome, bacterial diversity, *Cutibacterium*

## Abstract

**Introduction:** The human skin microbial composition is affected by age. Previous studies reported skin microbiome diversity shifts between elderly and significantly younger subjects. Some studies implied that menopausal status, which is inherently linked to age, could be associated with changes in skin microbial compositions. Nevertheless, the influence of menopausal status on skin microbiome profiles while minimizing the impact of aging-associated changes in skin parameters still needs further clarification.

**Methods:** We performed an observational study on healthy Caucasian female volunteers, which were grouped according to their pre- or postmenopausal status. Bacterial community structures on facial skin were analyzed using 16S rRNA gene sequencing. Cutometer^®^ measurements were performed to evaluate aging-associated changes in facial skin biophysical properties.

**Results:** The relative abundance of the lipophilic *Cutibacterium* genus was decreased, and bacterial diversity was increased in skin samples of postmenopausal volunteers. The mean age difference between examined groups in this study was 12.4 years only. Accordingly, Cutometer^®^ measurements revealed no differences in aging-associated skin biophysical parameters between pre- and postmenopausal groups. Consequently, no correlation was detected between Shannon diversity and measured age-dependent biomechanical properties of facial skin.

**Discussion:** These findings are in line with previous studies, which investigated the wide-ranging impact of chronological aging on skin microbial communities. However, this work reports for the first time a direct association between menopausal status and facial microbiomes on skin of similarly aged study participants, and hence uncouples aging-associated skin biophysical parameters, such as viscoelastic properties, from the equation. These findings open avenues for the development of microbiome-targeting strategies for treatment of menopause-associated skin disorders.

## 1 Background

Menopause is a natural phase in the life of women and marked by the end of reproductive years (NCBI, 1996). The median menopausal age among Caucasian women from industrialized countries is 51 years (Gold, 2011) and preceded by a perimenopausal time, during which menstrual cycles become irregular. Once the last menstrual period has ceased for at least 12 months, the postmenopausal life stage is reached (Brambilla and McKinlay, 1989).

Physical symptoms experienced during the different menopausal stages are diverse and severity is variable among individuals. Skin and hair menopausal symptoms are mainly caused by a decline in circulating blood estrogen levels (Thornton, 2013), and include dryness and pruritus, epidermal and dermal thinning, increased sagging and wrinkle formation, poor wound healing, diminished vascularity, as well as reduced growth, quality and density of hair on scalp, and unwanted growth of hair on facial sites (Zouboulis et al., 2022).

After menopause, sebaceous gland activity gradually declines (Pochi et al., 1979). This decreased sebum production on skin not only contributes to an accelerated trans-epidermal water loss (Conti et al., 1995), but also impacts composition of skin-resident microorganisms by limiting the availability of sebaceous lipids as a nutritional source (Oh et al., 2012). Indeed, skin regions with high sebaceous gland activity, such as forehead, scalp, chest and upper back support colonization by lipophilic skin microbial community components such as *Malassezia* (Jo et al., 2016), *Cutibacterium* genera and most species belonging to the *Corynebacterium* genus (Oh et al., 2012; von Graevenitz et al., 2006).

Skin is a diverse ecosystem, as it consists of distinct dry, moist and oily microenvironments. Its structure is composed of different specialized cell types, and possesses appendages like hair follicles, sebaceous glands and sweat glands. As such, skin offers a diverse and rich environment for population by a myriad of microorganisms each evolutionary adapted to inhabit one of the many skin niches (Byrd et al., 2018). Recently, it has become evident, that the skin microbial community, consisting of bacterial, archaeal, fungal and arthropodal organisms, including viruses, contributes actively to skin homeostasis and health (Sfriso et al., 2020). Dysbiotic changes in the skin microbiota are associated with skin and scalp disorders, such as eczema, acne, dandruff and chronic wounds (Byrd et al., 2018).

While substantial efforts have been made to better understand connections between shifts in skin microbial community structures and chronological age (Luna, 2020), investigations into determining direct impacts of menopausal statuses, even though inherently linked to age, were neglected so far. On the contrary, research into menopause-associated changes in oral, gut and vaginal microbiome are well advanced (Brotman et al., 2014; Vieira et al., 2017).

Changes in skin microbiome diversity and composition at different taxonomic levels linked to ageing were described previously in several female cohort studies. However, mean age differences between examined old and young age groups in these studies were between 32 and 39 years (Shibagaki et al., 2017; Somboonna et al., 2017; Jugé et al., 2018; Kim et al., 2019; Kim et al., 2022; Zhou et al., 2023), or examined age groups were multimodal distributed (Howard et al., 2022; Russo et al., 2023). Ageing is a complex and multifactorial process, and the composition of the skin microflora can be influenced by age-dependent exposure time spans to environmental stressors and intrinsic factors (Khmaladze et al., 2020), such as solar UV irradiation, particulate matter, cosmetic products, climate, nutritional ingredients, as well as individual genetic background, gender, menopause-associated hormonal changes and immune-senescence (Cisneros et al., 2022). To shed light on a potential direct association between skin microbiome compositions and menopausal status, and importantly to minimize above age-dependent confounding effects, we designed and executed an appropriate observational human study. Specifically, bacterial profiles were compared between pre- and postmenopausal facial skin sites of female volunteers with similar ages.

## 2 Materials and methods

### 2.1 Study background

Unpublished data on facial skin microbiome profiles and skin biophysical measurements were extracted from a previously performed single-center study (Campiche and Pascucci, 2023), which investigated the impact of a combination of a *Nannochloropsis oculata* microalgae extract and high-performance polysaccharide (Pullulan) [PEPHA^®^-TIGHT CB] on skin texture, firmness and elasticity of female volunteers. As described therein, female volunteers applied over the course of 7 days twice daily either an active [PEPHA^®^-TIGHT CB] or a placebo formulation on their face. Briefly, this study showed that application of [PEPHA^®^-TIGHT CB] provided an instant facial skin care benefit compared to usage of a placebo formulation. Compositions of used formulations can be retrieved from (Campiche and Pascucci, 2023).

### 2.2 Study participants

Healthy, Caucasian female volunteers, aged 40–65, were grouped according to their pre- (*n* = 14, average age 48.1 ± 3 years) or postmenopausal (*n* = 30, average age 60.6 ± 4 years) status, defined by whether menstrual cycles were regular or whether the last menstrual period occurred at least 12 months ago, respectively. Perimenopausal and menopausal individuals were excluded from this study, except where explicitly mentioned. In order to minimize confounding factors that are known to impact skin microbiome compositions, strict exclusion criteria for selection of study participants included, but were not limited to, being in the course of immunosuppressive treatment, under long-term treatment, in particular with products containing aspirin and derivatives, anti-inflammatory, antibiotics, antihistamines, steroid hormones, such as those being integral components of hormonal replacement therapies (HRT), beta blockers and/or desensitization drugs. Furthermore, subjects having a skin disease, having facial skin recently exposed to sunlight (natural or artificial) within 2 weeks preceding the inclusion, premenopausal subjects being pregnant, and subjects having applied anti-wrinkle, anti-aging and firming products during the 4 weeks preceding the starting day of the study, were not allowed to participate.

### 2.3 Sampling of facial skin microbial DNA

Samples were obtained in accordance with the Declaration of Helsinki. All study volunteers had given their informed consent to participate before enrolment. Skin samples were collected in France (Lyon) from forehead and from randomly chosen either left or right cheek sites using DNA/RNA shield swabs (Zymo Research, Cat #R1106). Samples were taken on day 0 and day 7, totaling 4 samples from each subject. The volunteers were instructed not to wash their faces on the day of sampling. The swab was premoistened in sterile PBS prior to rubbing the skin area of 2 × 2 cm (4 cm^2^ surface area) for 30 s in different directions. Sample material was placed into DNA/RNA shield storage tubes (Zymo Research, Cat #R1106) and stored at −80°C until processing.

### 2.4 16S rRNA gene sequencing and data analysis

DNA was extracted according to standard protocols, Illumina 16S rRNA gene amplicon libraries were generated and sequenced at BaseClear B.V. as described previously (Deyaert et al., 2023).

In short, barcoded amplicons from the V3-V4 region of 16S rRNA genes were generated using a 2-step PCR with the primers 16S‐341F and 16S‐785R. In order to decrease potential biases associated with PCR amplification using 16S rRNA gene V3-V4 primers (Meisel et al., 2016) during this skin microbiome study, Phusion high-fidelity polymerase was used to reduce errors in sequence amplification (Sze and Schloss, 2019). The libraries were barcoded, multiplexed, and sequenced on an Illumina MiSeq machine with a paired-end 300 cycles protocol and indexing. Illumina sequencing data were quality checked and demultiplexed by BaseClear standards, and FASTQ files were generated. For data processing, paired-end sequence reads were merged into pseudoreads through sequence overlap. Chimeric pseudoreads were removed and remaining reads were aligned against the RDP database for bacterial organisms (Cole et al., 2014). Based on the alignment scores of the pseudoreads, the taxonomic depth of the lineage is based on the identity threshold of the rank; Species 99%, Genus 97%, Family 95%, Order 90%, Class 85%, Phylum 80%.

### 2.5 Measurement of skin elasticity

To evaluate aging-associated changes in facial skin biophysical properties of study participants, Cutometer^®^ measurements were performed according to standardized guidelines. Aberrant values were excluded from statistical analyses reported here. During the baseline visit, the viscoelastic properties of the forehead and cheek skin were evaluated using a Cutometer^®^ device, which measures the deformation of a cutaneous area submitted to a mechanical suction and its recovering power.

### 2.6 Statistical analysis

Preprocessing of abundance data was performed using MicrobiomeAnalyst 2.0 (Lu et al., 2023), while calculations and visualizations were performed in a Jupyter notebook in a Python v3.10.11 environment with the following relevant packages installed: Pandas 2.0.0, Numpy v1.23.5, Scipy v1.11.3, scikit-bio v0.5.9, sklearn v1.1.2, Seaborn v0.12.2, Matplotlib v3.7.1.

Diversity and richness were calculated using Shannon’s entropy and Chao1, respectively. Bray-Curtis divergence was used to compute the inter-sample distance. Principal Component Analysis (PCA) was performed on the obtained distance matrix. PERMANOVA (99999 permutations) and PERMDISP (999 permutations) testing was used to assess clustering differences between groups. Unless otherwise stated, non-parametric independent testing using Mann-Whitney *U* test was performed when comparing different subjects for measures within pre- and post-menopausal groups. Non-parametric paired testing using Wilcoxon test was performed when comparing subject dependent features. Original datasets are available in a publicly accessible repository: Sequence Read Archive (SRA) portal of NCBI under accession number PRJNA1079725.

## 3 Results

### 3.1 Facial skin of postmenopausal study participants is not measurably older than premenopausal skin

We designed and executed an observational study to investigate a potential attribution of menopausal status rather than differences in aging skin and the underlying skin biophysical properties to a shift in skin microbial community structures, allowing to minimize influences of other age-associated confounders. With average group ages of 48.1 ± 3 and 60.6 ± 4 years, respectively, representative pre- and postmenopausal study participants were specifically chosen to belong to an age range close to menopausal transition. The median age at menopause among Caucasian women from industrialized nations is 51 (Gold, 2011). Of note, the oldest participants in the premenopausal group were older than the youngest participants in the postmenopausal group (Supplementary Table S1).

**FIGURE 1:**
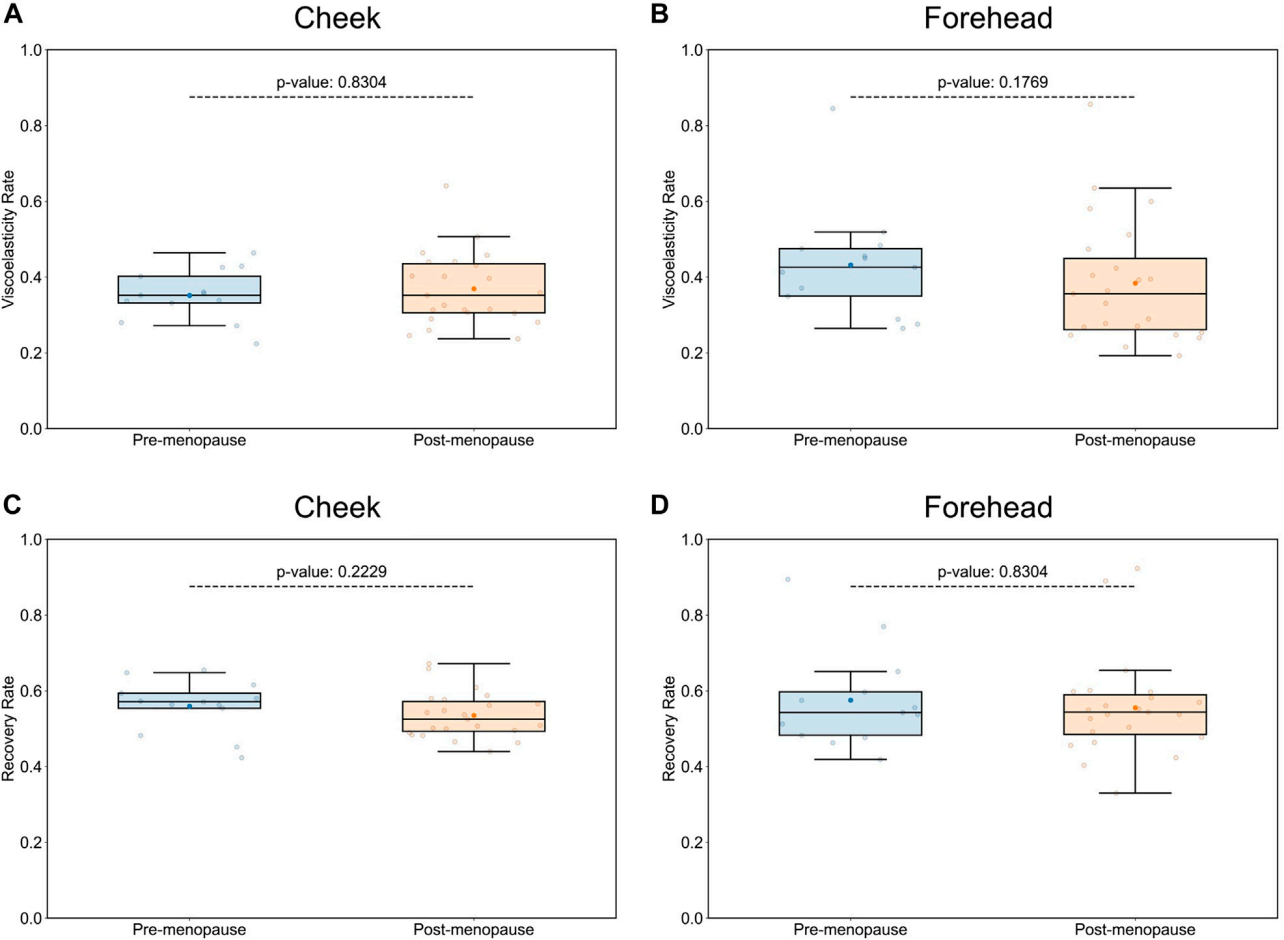
Comparison of skin biophysical properties between pre- and postmenopausal groups. Cutometer^®^ measurements did not reveal significant differences in aging-associated elastic properties of cheek and forehead skin, such as visco-elasticity **(A,B)**, or gross elasticity **(C,D)**, between pre- and post-menopausal volunteers. Each point represents the measured numerical variable, i.e., Cutometer^®^ reading for the indicated skin site of a study participant (*n* = 36; 13 for pre-, and 23 for postmenopausal subjects). Groups are color-coded according to their pre- or postmenopausal status. The box plots were generated using standard interval ranges (Q1 and Q3 for quartiles, 1.5 IQR for whiskers). Solid line within the box marks the median, the solid dot marks the mean, and the points beyond the whiskers are considered outliers. Respective *p*-values are indicated above the upper whiskers.

Assuming that age-dependent skin physiological parameters are critical determinants for microbial niche selection and population, we would not expect any significant changes in skin microbiome compositions between “younger” pre- and “older” postmenopausal groups, should their measurable skin biophysical conditions, such as viscoelastic properties, be similar. As previously reported, facial sagging is inherently linked with facial skin firmness, and hence is the most accurate predictor of chronological age (Flament et al., 2021), consistent across different ethnical groups (Voegeli et al., 2023). Given that the average age difference between the two examined groups was 12.4 years only, comparison of Cutometer^®^ readings between pre- and postmenopausal groups (Supplementary Table S1) revealed no significant differences in aging-associated skin elasticity and tiring effects (Figure 1). With the main discriminants between the examined study groups being their respective pre- or postmenopausal status and associated skin physiological parameters, we next examined genomic structures of facial skin bacterial communities.

### 3.2 Facial skin microbiome stability over the course of the study

To investigate an association between skin microbiome composition and menopausal status, we performed 16S rRNA gene amplicon sequencing of bacterial DNA isolated during two timepoints over the course of a week, separately from cheek and forehead skin sites. A total of 2′885′744 reads were analyzed with an average read count of 14′723 per sample, and the sequences were assigned to 5,854 operational taxonomic units (OTUs) based on ≥ 97% sequence identity (Supplementary Table S2). Skin microbiome profiling of each study participant twice during 1 week, on different facial skin sites and in dependence of topical application of the investigational cosmetic product, allowed not only to gain statistical power for comparative data analysis, but also to investigate temporal and intra-personal variability of skin microbiome profiles, as well as to explore the impact of specific extrinsic factors, such as cosmetic product use, on skin bacterial compositions: Topical application of either active or placebo formulations over the course of 1 week had no impact on skin bacterial compositions, as α- and β-diversities (measured by Shannon’s index and Bray-Curtis dissimilarity, respectively) assessed at the taxonomic bacterial genus level were not significantly different between placebo and verum groups after 7 days of applications (Supplementary Figure S1), and as such microbiome-friendliness can therefore be attributed to the formulations. In accordance with previous publications (Oh et al., 2016), skin microbial community structures on forehead and cheek were characterized by longitudinal stability, as α- and β-diversities were not significantly different between baseline sampling on day 0, and day 7 (Supplementary Figure S2). Given the temporal stability and resistance of skin-resident microbial compositions to tested active and placebo formulations, we decided to include all collected microbiome data sets for following analyses.

### 3.3 Microbiome compositions are different between pre- and postmenopausal facial skin

**FIGURE 2:**
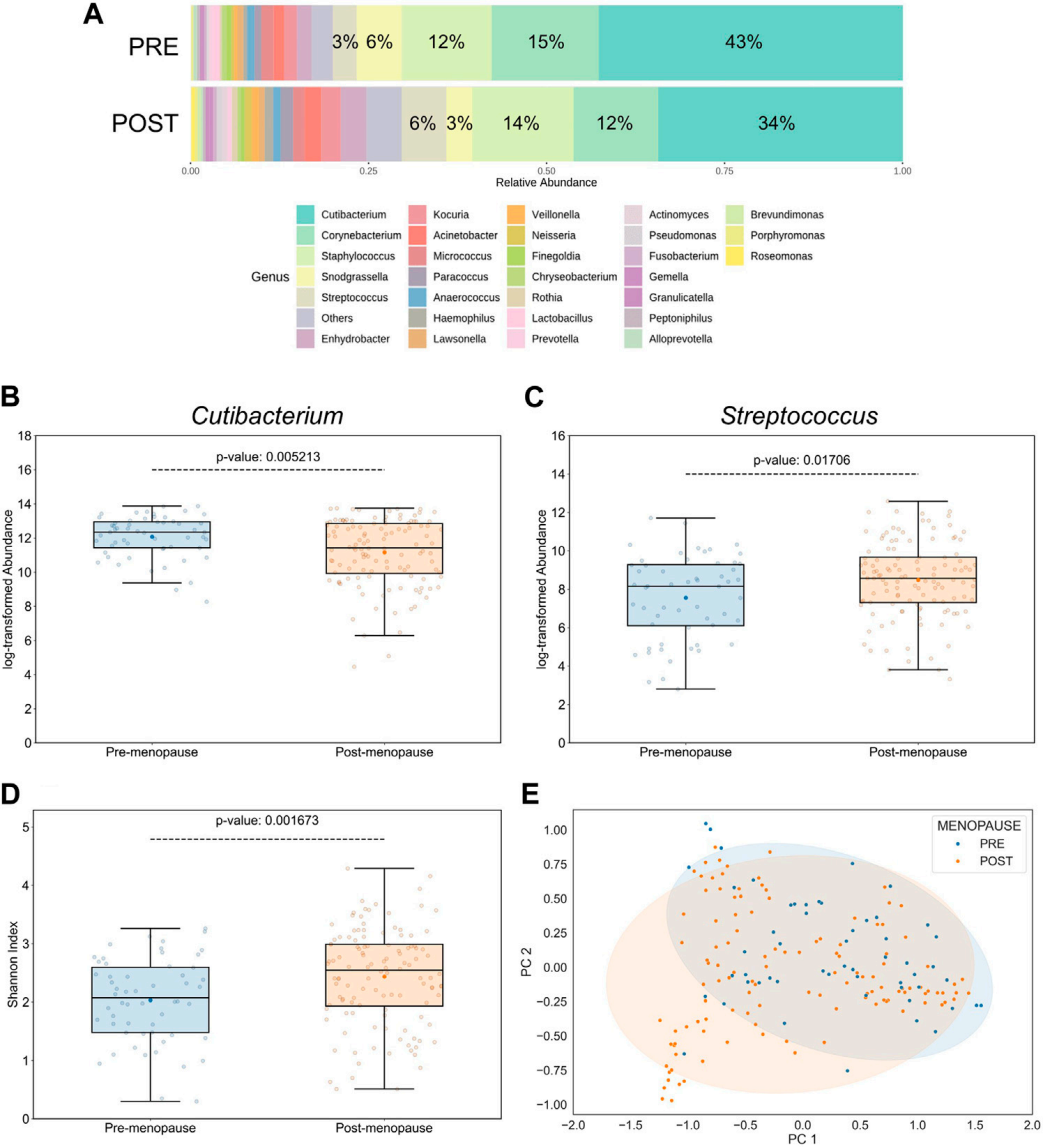
Effect of menopausal status on facial skin microbiome profiles. **(A)** Distribution of top 30 bacterial genera on pre- and postmenopausal facial skin. A total of 82 bacterial genera from 9 phyla were found in all samples. Of these, 5 predominant genera occupied >79% in the premenopausal group and >71% in the postmenopausal group. Small taxa with counts <10 were merged and labelled as “Others.” Mean relative abundances for each of the top 5 genera are displayed. **(B)** Relative abundances of *Cutibacterium* and **(C)** *Streptococcus* genera on facial skin sites of postmenopausal women are significantly lower and higher, respectively, than on premenopausal skin. **(D)** Genus-level bacterial diversity, measured by Shannon’s index, is higher on postmenopausal than on premenopausal facial skin sites. Boxplot features are described in Figure 1. Respective *p*-values are indicated above the upper whiskers. **(E)** Skin microbial composition is significantly different between the two menopausal statuses (PERMANOVA *p* = 0.00156, *r* ^2^ = 0.0432; PERMDISP *p* = 0.006). Ellipses indicate 95% confidence intervals. For analyses shown here, all collected skin samples were used (*n* = 176; 56 for pre-, and 120 for postmenopausal subjects).

According to taxonomical assignments, which were performed using the pipeline integrated within the MicrobiomeAnalyst 2.0 package (Lu et al., 2023), the 5 most abundant genera, comprising >79% of the microbiomes, were *Cutibacterium, Corynebacterium, Staphylococcus, Snodgrassella*, and *Streptococcus* (Figure 2A). Relative abundances of bacterial genera were differentially distributed on facial skin between pre- and postmenopausal volunteers. For example, the lipophilic *Cutibacterium* genus was not only the most proportionally represented genus on skin of all study participants, but also significantly more abundant in the premenopausal group (Figure 2B). On the other hand, relative abundances of OTUs affiliated to the *Streptococcus* genus, which belongs to the *Bacillota* phylum with preference for skin sites with low sebaceous gland activity, such as infant skin (Luna, 2020), was found to be significantly increased in the postmenopausal group (Figure 2C). Moreover, the *Streptococcus* genus was previously shown to negatively correlate with facial sebaceous gland area (Howard et al., 2022). This finding aligns well with previously published data, that skin sites enriched with sebaceous lipids attract lipophilic microorganisms (Byrd et al., 2018). Other bacterial genera, whose relative abundances were significantly affected by menopausal statuses, are listed in Supplementary Table S3.

### 3.4 Bacterial diversity on facial skin is linked to menopausal status

Intriguingly, the α-diversity (Shannon’s index) and β-diversity (Bray-Curtis dissimilarity), assessed at the genus taxonomic level, were significantly different between pre- and postmenopausal groups at sampled facial skin sites (Figures 2D, E). Specifically, bacterial α-diversity was significantly increased in skin samples obtained from postmenopausal volunteers, a finding that is in line with previous reports stating higher α-diversity on ageing skin compared to significantly younger skin of females (Shibagaki et al., 2017; Jugé et al., 2018; Kim et al., 2022; Zhou et al., 2023). The statistical significance regarding differences in menopause-associated α- and β-diversities was maintained even after mathematical removal of the most abundant *Cutibacterium* genus from the equation (Supplementary Figure S3), suggesting that evenness deflation of the samples caused by dominance of *Cutibacterium* is not the only driver of bacterial diversification.

### 3.5 Skin bacterial diversity does not correlate with aging-associated skin biophysical properties

The measured skin parameters, such as elasticity and recovery rate, were not significantly different between pre- and postmenopausal groups in our study (Figure 1), whereas skin microbiome profiles were affected by the respective menopausal statuses (Figure 2). As expected, no significant associations between biophysical parameters and bacterial diversity on skin could be detected (Supplementary Figure S4), suggesting that the observed shift in skin microbial profiles can be attributed directly to menopausal status rather than to differences in aging-associated viscoelastic properties.

### 3.6 Bacteria residing on forehead drive menopause-associated facial microbiome diversification

**FIGURE 3:**
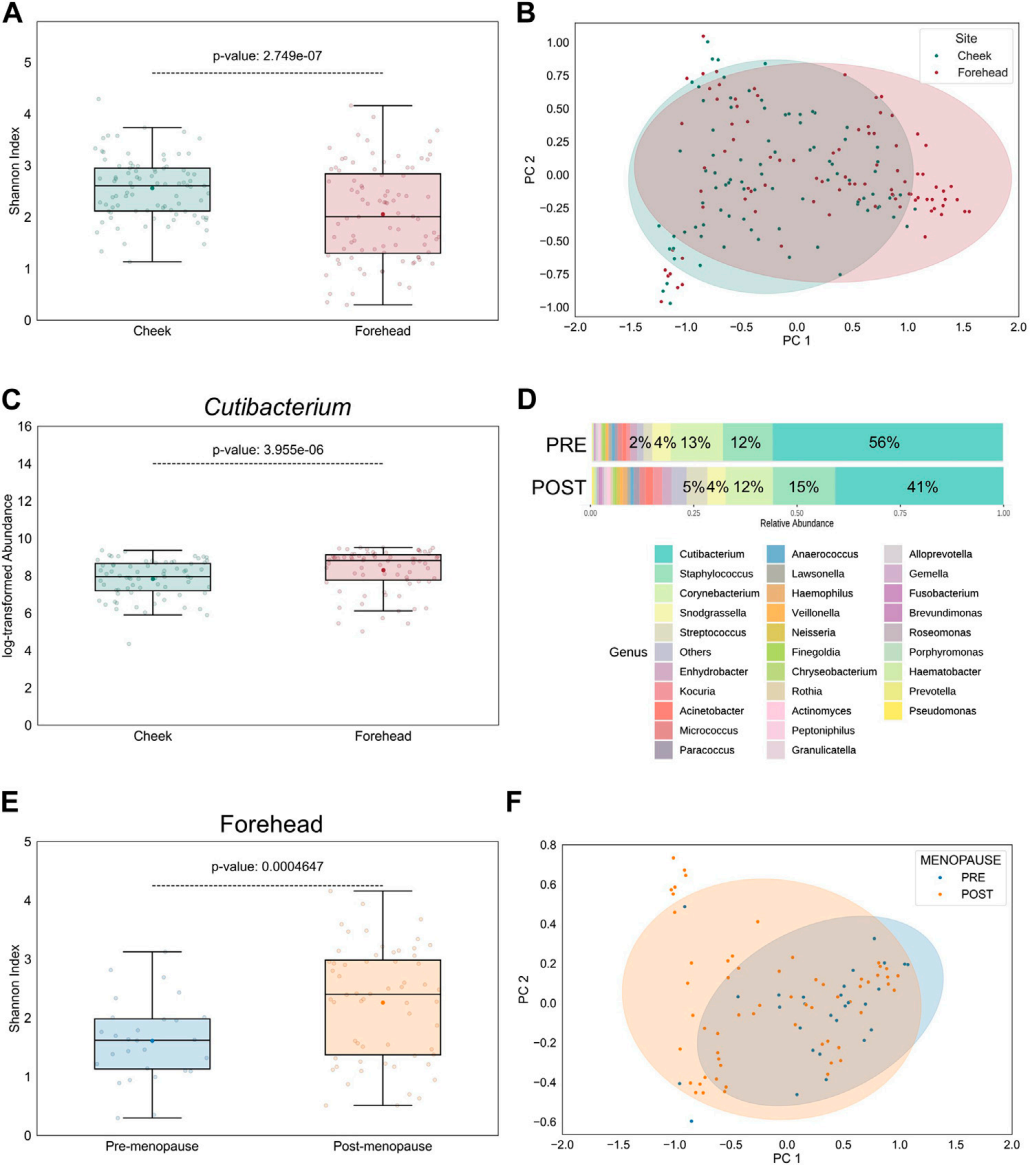
Intra-personal variation in bacterial diversity between pre- and postmenopausal facial skin sites. **(A)** Bacterial diversity at taxonomic genus-level is significantly higher on cheek than on forehead skin. Boxplot features are described in Figure 1. **(B)** Skin microbial composition is significantly dissimilar between the two skin sites (PERMANOVA *p* = 8E-5, *r* ^2^ = 0.0652; PERMDISP *p* = 0.65). Ellipses indicate 95% confidence intervals. For analyses shown in **(A,B)**, all collected skin samples were used (*n* = 176; equally distributed for both skin sites). **(C)** Relative abundance of the lipophilic *Cutibacterium* genus on cheek is significantly lower than on forehead (*n* = 144; equally distributed for both skin sites). **(D)** Distribution of top 30 bacterial genera on forehead skin of pre- and postmenopausal volunteers. A total of 77 bacterial genera from 9 phyla were found in all samples. Of these, 5 predominant genera occupied >87% in the premenopausal group and >77% in the postmenopausal group. Small taxa with counts <10 were merged and labelled as “Others.” Mean relative abundances for each of the top 5 genera are displayed. **(E)** Bacterial diversity is higher on post-than on premenopausal forehead skin. **(F)** Skin microbial composition on forehead is significantly different between the examined groups (PERMANOVA *p* = 0.0067, *r* ^2^ = 0.08; PERMDISP *p* = 0.008). For analyses shown in **(E,F)**, all collected skin samples from forehead were used (*n* = 88; 28 for pre-, and 60 for postmenopausal subjects).

The topography of skin largely impacts the distribution of microbes, indicating the presence of selection pressure: Sebaceous skin, found, for example, on forehead, contains the lowest microbial diversity, while comparably drier sites, such as cheek skin, favors a broader diversity (Mukherjee et al., 2016). Accordingly, in our study the bacterial α- and β-diversities were significantly higher in cheek than in forehead samples and significantly dissimilar between the skin sites, respectively (Figures 3A, B). Aligning with previous findings (Grice et al., 2009; Findley et al., 2013), the relative abundances of lipophilic microbes, such as those belonging to the *Cutibacterium* genus, were significantly increased on forehead (Figure 3C), a skin site that is characterized by higher sebaceous gland density compared to cheek skin (Thody and Shuster, 1989).

Given these intra-personal variabilities between facial skin sites we wondered whether the observed menopause-regulated shift in facial skin microbiome profiles (Figure 2) was powered by forehead- and/or cheek-resident bacterial communities. Indeed, differences in bacterial genera distributions (not shown), as well as α- and β-diversities, on cheek skin between pre- and postmenopausal volunteers were reduced (Supplementary Figures S5A, B), compared to forehead skin of the same volunteers (Figures 3D–F). Nevertheless, Chao1 index was significantly increased in cheek skin samples of postmenopausal volunteers (Supplementary Figure S5C), suggesting that rare taxa richness, rather than their even distribution, was the main driver for the increased bacterial diversification. The difference in menopause-associated α-diversity remained statistically significant even after mathematical removal of the *Cutibacterium* genus from forehead samples (Supplementary Figure S6A), whereas bacterial communities became similar (Supplementary Figure S6B).

### 3.7 Menstrual cycle regularity affects facial skin microbiome profiles

A small subject number (*n* = 4) reported irregular menstrual cycles and as such was classified as perimenopausal and excluded from the main study reported above. Nevertheless, inclusion of this group and subsequent direct comparison with pre- and postmenopausal volunteers revealed that respective bacterial genus-level α-diversities as measured by Shannon’s index in samples were significantly different (Supplementary Figure S7). In agreement with a previous report stating that microbial diversity was reduced on facial skin sites of female volunteers with self-evaluated irregular menstrual periods (Ma et al., 2023), Shannon’s index of perimenopausal skin samples was significantly lower than from pre- and postmenopausal subjects in our study (Supplementary Figure S7).

## 4 Discussion

It is not trivial to distinguish between impacts of natural aging factors and menopausal symptoms on the female body, including hair and skin. To investigate the direct influence of menopause, resulting from loss of ovarian function, on facial skin microbiomes, we designed and executed an appropriate observational pilot study allowing to reduce potential influences of confounders associated with aging. Indeed, ageing is a complex and multifactorial process, and several age-dependent confounding factors can leave footprints on skin microbial community structures.

This study revealed an increased bacterial diversity on facial skin sites of postmenopausal subjects, a finding that is in line with previous reports, which stated a broader variety of microbes on aging skin (Luna, 2020).

The skin microbial community is characterized by being longitudinally stable over several years (Oh et al., 2016). Indeed, previous reports only detected shifts in skin microbiome community structures after comparison of healthy groups with broad mean age differences (Jo et al., 2016; Jugé et al., 2018; Dimitriu et al., 2019; Howard et al., 2022; Kim et al., 2022) and correlations to menopausal statuses were only implied. Our herewith presented results describing menopausal status-dependent changes in skin microbial diversity and composition can be substantiated from these prior studies by the fact that the average age difference between the two examined groups was 12.4 years only, and, importantly, did not significantly impact measured ageing-associated skin viscoelastic properties (Figure 1). Therefore, we propose that the main differentiating factor between the two examined groups with regards to influence on skin microbial profiles was a hormone-driven impact associated with their menopausal status. For example, androgen-stimulated sebum production in skin of postmenopausal women is significantly reduced compared to men of similar age (Pochi et al., 1979). Indeed, the decrease in relative abundance of the lipophilic *Cutibacterium* genus on postmenopausal skin, which is characterized by dry and moist properties (Grice and Segre, 2011), may have allowed other bacterial genera, such as *Streptococcus*, to populate the niche, inflate evenness and consequently increase observed bacterial diversity.

One limitation of the study is that expected variations in skin surface parameters, such as pH, sebum and hydration levels were not measured. Hormones drive not only sebum but also sweat gland development, thereby impacting availability of key nutrients (Townsend and Kalan, 2023) and physical conditions required by microorganisms to proliferate. Indeed, stratum corneum acidification and skin hydration are impaired in moderately aged human skin (Choi et al., 2007; Man et al., 2009). Particularly water-binding hyaluronic acid concentrations are diminished in estrogen-deficient skin, thereby negatively affecting the capability of postmenopausal skin to remain hydrated (Patriarca et al., 2013). Sebum and hydration levels indeed seem to be closely correlated with the nature of microbial compositions (Akaza et al., 2023), as these parameters were also shown to accurately predict facial skin microbiome diversity (Mukherjee et al., 2016).

The proportional abundance of *Lactobacillus* was significantly reduced on postmenopausal skin (Supplementary Table S3). Interestingly, the menopausal decline in estrogen levels results in decreased vaginal epithelial synthesis of glycogen (Calleja-Agius and Brincat, 2015), which is required for proliferation of *Lactobacillus*. A depletion of this genus may not only contribute to reduced acidification of skin, but also allow other species to populate the same niche due to reduced production of antimicrobial and anti-inflammatory metabolites by lactobacilli (Delanghe et al., 2021).

Moreover, a recent study showed that estrogens increase the gut microbiome diversity and directly upregulate microbial enzymes involved in estrogen metabolism, such as β-glucuronidase (Lephart and Naftolin, 2022). More research is required to reveal whether skin surface hormones are capable of directly modulating skin microbiome community structures and respective microbial enzymatic activities, and which menopause-associated skin physiological changes can be attributed individually or collectively to adaptations of skin microbial profiles.

In the first 5 years after menopause, skin collagen content decreases by up to 30%, with stronger correlation between collagen degradation rate and estrogen deficiency rather than chronological age (Brincat et al., 1985; Affinito et al., 1999). Additionally, the menopause-associated reduction of skin epidermal thickness (Brincat et al., 1987) would be expected to reduce skin viscoelasticity and recovery rate of postmenopausal participants in our study. However, in contrast to a recent report (Zhou et al., 2023), age-related facial viscoelastic properties were similar between pre- and postmenopausal groups (Figure 1), and consequently no correlation with facial microbiome diversity was detected (Supplementary Figure S4), which suggests that menopausal status had a more significant impact on composition of the facial microflora than aging-associated skin biophysical properties. The narrow mean age difference of only 12.4 years between pre- and postmenopausal volunteers in our study, as compared to 34 years (Zhou et al., 2023), may explain why no dissimilarities in biophysical skin aging measurements were detected. Alternatively, microbes may have adapted faster to menopause-associated skin physiological changes, such as variations in sebum production or collagen density, than skin biophysical properties. A follow-up study involving pre- and postmenopausal volunteers with no mean age differences between the groups will be required to ultimately evaluate the contribution of menopausal status on skin community structures.

Genetic and metabolic pathways of skin-resident microorganisms may accelerate physiological skin aging processes through production of enzymes and bioactive molecules involved in collagen degradation or protein glycation (Alkema et al., 2021; Zhou et al., 2023). Our study contradicts this hypothesis, given that cheek and forehead skin of postmenopausal volunteers was not measurably older, however skin microbial community structures were significantly impacted by menopausal status. Nevertheless, for the same reasons as above, it could be that the potential long-term impact of microbial metabolic activity on skin aging processes was lagging the underlying microbial niche adaptations.

The main results and conclusions described in our study were derived from comparative analyses between strictly defined pre- and postmenopausal groups. Inclusion of female subjects with irregular menstrual cycles, conventionally categorized either as peri- or menopausal, showed that facial microbiome diversity was significantly lower than in samples obtained from pre- and postmenopausal skin (Supplementary Figure S7), as previously reported for Chinese women (Ma et al., 2023). These findings are interesting, even though a confirmation with higher subject number is required, in that inclusion of volunteers with irregular menstrual periods inaccurately in either pre- or postmenopausal groups may significantly impact respective microbial diversity calculations. Inappropriate inclusion of transmenopausal subjects in postmenopausal groups, as well as other confounding age-dependent and extrinsic factors may explain, why a minority of previous studies detected a trend towards higher bacterial diversity on face of younger volunteers (Kim et al., 2019; Russo et al., 2023). Lastly, given that the average age of volunteers in our peri/menopausal group (45.8 ± 4 years) was similar to the directly compared premenopausal group (48.1 ± 3 years), further supports our hypothesis that the impact of menopausal-driven hormonal changes on facial microbiome composition may be more significant than age-dependent skin physiological adaptations. Further research will be required to understand the mechanisms by which menopause-associated physiological parameters interact with the skin microbial flora.

In summary, our results suggest that using menopausal status, rather than chronological age as the main discriminant for grouping of female cohorts, may provide more accurate results from observational studies. Furthermore, our study revealed not only significant intra-personal differences in skin microbiome compositions between facial skin sampling sites, but also that conclusions drawn from studies investigating extrinsic and intrinsic impacts, such as menopausal status, on microbial community structure shifts strongly depend on facial skin sampling sites.

### 4.1 Conclusion

Worldwide life expectancy is steadily increasing, and the number of postmenopausal women is expected to reach 1.2 billion by 2030 (Afshari et al., 2020). This rapidly growing population now lives one-third of their lifetime in a state of estrogen deficiency, hence there is an unmet need to expand our knowledge on the role of skin microbiome in postmenopausal health. Several gut and vaginal microbiome-targeting therapies exist for treatment of menopause-associated burdens (Park et al., 2023). However, the potential of skin microbiome-modulating strategies to manage skin disorders associated with menopausal states is waiting to be exploited.

We hypothesize that an increased bacterial diversity and potentially associated dysbiosis in the skin microbiome may at least partially contribute to the development of skin disorders experienced by trans- and postmenopausal women. Thus, targeted microbiome-based interventions aiming at rebalancing the skin microbiome composition may provide alternative approaches to traditional symptom reduction or more drastic treatments, such as hormonal replacement therapy (HRT), and hence reduce associated side effects. HRT is often prescribed to women to alleviate adverse physiological changes experienced during menopause. While the effects of HRT on gut and vaginal microbial community structures have been investigated (Dothard et al., 2023), impacts on skin microflora are not known. Given that HRT stimulates sebum secretion (Sator et al., 2001), we would expect the skin of postmenopausal patients using HRT to be repopulated by lipophilic microorganisms, such as *Cutibacterium acnes*, followed by a decrease in microbiome diversity due to evenness deflation of competing microbial components.

Future studies into better understanding mechanisms of interactions between circulating sex hormonal levels and the skin microbiome are required for the development of non-hormonal, microbiome-targeting therapeutics for treatment of skin and hair menopausal symptoms.

## Data Availability

Original datasets are available in a publicly accessible repository: Sequence Read Archive (SRA) portal of NCBI under accession number PRJNA1079725.

## Supplementary material

SUPPLEMENTARY TABLE S1 Metadata.

SUPPLEMENTARY TABLE S2 OTU table.

SUPPLEMENTARY TABLE S3 Genus abundance differences.

## References

[B1] Affinito P. Palomba S. Sorrentino C. Di Carlo C. Bifulco G. Arienzo M. P. (1999). Effects of postmenopausal hypoestrogenism on skin collagen. Maturitas 33 (3), 239–247. 10.1016/s0378-5122(99)00077-8 10656502

[B2] Afshari F. Bahri N. Sajjadi M. Mansoorian M. Tohidinik H. (2020). Menopause uncertainty: the impact of two educational interventions among women during menopausal transition and beyond. Menopause Review/Przegląd menopauzalny. 19 (1), 18–24. 10.5114/pm.2020.95305 PMC7258369 32508552

[B3] Akaza N. Takasaki K. Matsudaira T. Usui A. Iijima A. Miura S. (2023). Relationship between skin fungal and bacterial microbiomes and skin pH. Int. J. Cosmet. Sci. 45 (3), 362–372. 10.1111/ics.12842 36752033

[B4] Alkema W. Boekhorst J. Eijlander R. T. Schnittger S. De Gruyter F. Lukovac S. (2021). Charting host-microbe co-metabolism in skin aging and application to metagenomics data. PloS one 16 (11), e0258960. 10.1371/journal.pone.0258960 34758050 PMC8580226

[B5] Brambilla D. J. McKinlay S. M. (1989). A prospective study of factors affecting age at menopause. J. Clin. Epidemiol. 42 (11), 1031–1039. 10.1016/0895-4356(89)90044-9 2809660

[B6] Brincat M. Versi E. Moniz C. F. Magos A. de Trafford J. Studd J. W. (1987). Skin collagen changes in postmenopausal women receiving different regimens of estrogen therapy. Obstetrics Gynecol. 70 (1), 123–127. 10.1016/0378-5122(87)90045-4 3601260

[B7] Brincat M. Moniz C. J. Studd J. W. W. Darby A. Magos A. Emburey G. (1985). Long-term effects of the menopause and sex hormones on skin thickness. BJOG Int. J. Obstetrics Gynaecol. 92 (3), 256–259. 10.1111/j.1471-0528.1985.tb01091.x 3978054

[B8] Brotman R. M. Shardell M. D. Gajer P. Fadrosh D. Chang K. Silver M. I. (2014). Association between the vaginal microbiota, menopause status, and signs of vulvovaginal atrophy. Menopause (New York, NY) 21 (5), 450–458. 10.1097/GME.0b013e3182a4690b PMC3994184 24080849

[B9] Byrd A. L. Belkaid Y. Segre J. A. (2018). The human skin microbiome. Nat. Rev. Microbiol. 16 (3), 143–155. 10.1038/nrmicro.2017.157 29332945

[B10] Calleja-Agius J. Brincat M. P. (2015). The urogenital system and the menopause. Climacteric 18 (1), 18–22. 10.3109/13697137.2015.1078206 26366796

[B11] Campiche R. Pascucci F. (2023). Pepha-tight study: an instant lift for skin through the power of algae. SOFW 149 (3), 26–31.

[B12] Choi E. H. Man M. Q. Xu P. Xin S. Liu Z. Crumrine D. A. (2007). Stratum corneum acidification is impaired in moderately aged human and murine skin. J. investigative dermatology 127 (12), 2847–2856. 10.1038/sj.jid.5700913 17554364

[B13] Cisneros B. García-Aguirre I. Unzueta J. Arrieta-Cruz I. González-Morales O. Domínguez-Larrieta J. M. (2022). Immune system modulation in aging: molecular mechanisms and therapeutic targets. Front. Immunol. 13, 1059173. 10.3389/fimmu.2022.1059173 36591275 PMC9797513

[B14] Cole J. R. Wang Q. Fish J. A. Chai B. McGarrell D. M. Sun Y. (2014). Ribosomal Database Project: data and tools for high throughput rRNA analysis. Nucleic acids Res. 42, D633–D642. 10.1093/nar/gkt1244 24288368 PMC3965039

[B15] Conti A. Schiavi M. E. Seidenari S. (1995). Capacitance, transepidermal water loss and causal level of sebum in healthy subjects in relation to site, sex and age. Int. J. Cosmet. Sci. 17 (2), 77–85. 10.1111/j.1467-2494.1995.tb00111.x 19250473

[B16] Delanghe L. Spacova I. Van Malderen J. Oerlemans E. Claes I. Lebeer S. (2021). The role of lactobacilli in inhibiting skin pathogens. Biochem. Soc. Trans. 49 (2), 617–627. 10.1042/BST20200329 33704415

[B17] Deyaert S. Moens F. Pirovano W. van den Bogert B. Klaassens E. S. Marzorati M. (2023). Development of a reproducible small intestinal microbiota model and its integration into the SHIME®-system, a dynamic *in vitro* gut model. Front. Microbiol. 13, 1054061. 10.3389/fmicb.2022.1054061 37008301 PMC10063983

[B18] Dimitriu P. A. Iker B. Malik K. Leung H. Mohn W. W. Hillebrand G. G. (2019). New insights into the intrinsic and extrinsic factors that shape the human skin microbiome. mBio 10 (4), 008399–e919. 10.1128/mBio.00839-19 PMC6606800 31266865

[B19] Dothard M. I. Allard S. M. Gilbert J. A. (2023). The effects of hormone replacement therapy on the microbiomes of postmenopausal women. Climacteric J. Int. Menopause Soc. 26 (3), 182–192. 10.1080/13697137.2023.2173568 37051868

[B20] Findley K. Oh J. Yang J. Conlan S. Deming C. Meyer J. A. (2013). Topographic diversity of fungal and bacterial communities in human skin. Nature 498 (7454), 367–370. 10.1038/nature12171 23698366 PMC3711185

[B21] Flament F. Abric A. Adam A. S. (2021). Evaluating the respective weights of some facial signs on perceived ages in differently aged women of five ethnic origins. J. Cosmet. dermatology 20 (3), 842–853. 10.1111/jocd.13612 32649786

[B22] Gold E. B. (2011). The timing of the age at which natural menopause occurs. Obstetrics Gynecol. Clin. N. Am. 38 (3), 425–440. 10.1016/j.ogc.2011.05.002 PMC3285482 21961711

[B23] Grice E. A. Kong H. H. Conlan S. Deming C. B. Davis J. Young A. C. (2009). Topographical and temporal diversity of the human skin microbiome. Sci. (New York, NY) 324 (5931), 1190–1192. 10.1126/science.1171700 PMC2805064 19478181

[B24] Grice E. A. Segre J. A. (2011). The skin microbiome. Nat. Rev. Microbiol. 9 (4), 244–253. 10.1038/nrmicro2537 21407241 PMC3535073

[B25] Howard B. Bascom C. C. Hu P. Binder R. L. Fadayel G. Huggins T. G. (2022). Aging-associated changes in the adult human skin microbiome and the host factors that affect skin microbiome composition. J. investigative dermatology 142 (7), 1934–1946.e21. 10.1016/j.jid.2021.11.029 34890626

[B26] Jo J. H. Deming C. Kennedy E. A. Conlan S. Polley E. C. Ng W. I. (2016). Diverse human skin fungal communities in children converge in adulthood. J. investigative dermatology 136 (12), 2356–2363. 10.1016/j.jid.2016.05.130 PMC5687974 27476723

[B27] Jugé R. Rouaud-Tinguely P. Breugnot J. Servaes K. Grimaldi C. Roth M. P. (2018). Shift in skin microbiota of Western European women across aging. J. Appl. Microbiol. 125 (3), 907–916. 10.1111/jam.13929 29791788

[B28] Khmaladze I. Leonardi M. Fabre S. Messaraa C. Mavon A. (2020). The skin interactome: a holistic "Genome-Microbiome-Exposome" approach to understand and modulate skin health and aging. Clin. Cosmet. investigational dermatology 13, 1021–1040. 10.2147/CCID.S239367 PMC7769076 33380819

[B29] Kim H.-J. Kim J. J. Myeong N. R. Kim T. Kim D. An S. (2019). Segregation of age-related skin microbiome characteristics by functionality. Sci. Rep. 9 (1), 16748. 10.1038/s41598-019-53266-3 31727980 PMC6856112

[B30] Kim H.-J. Oh H. N. Park T. Kim H. Lee H. G. An S. (2022). Aged related human skin microbiome and mycobiome in Korean women. Sci. Rep. 12 (1), 2351. 10.1038/s41598-022-06189-5 35149745 PMC8837753

[B31] Lephart E. D. Naftolin F. (2022). Menopause and the skin: old favorites and new innovations in cosmeceuticals for estrogen-deficient skin. Dermatology Ther. 12 (7), 53–69. 10.1007/s13555-020-00468-7 PMC7859014 33242128

[B32] Lu Y. Zhou G. Ewald J. Pang Z. Shiri T. Xia J. (2023). MicrobiomeAnalyst 2.0: comprehensive statistical, functional and integrative analysis of microbiome data. Nucleic acids Res. 51 (W1), W310–W318. 10.1093/nar/gkad407 37166960 PMC10320150

[B33] Luna P. C. (2020). Skin microbiome as years go by. Am. J. Clin. dermatology 21 (1), 12–17. 10.1007/s40257-020-00549-5 PMC7584528 32910437

[B34] Ma L. Jiang H. Han T. Shi Y. Wang M. Jiang S. (2023). The menstrual cycle regularity and skin: irregular menstrual cycle affects skin physiological properties and skin bacterial microbiome in urban Chinese women. BMC women's health 23 (1), 292. 10.1186/s12905-023-02395-z 37259058 PMC10230734

[B35] Man M. Q. Xin S. J. Song S. P. Cho S. Y. Zhang X. J. Tu C. X. (2009). Variation of skin surface pH, sebum content and stratum corneum hydration with age and gender in a large Chinese population. Skin Pharmacol. physiology 22 (4), 190–199. 10.1159/000231524 PMC2836947 19648780

[B36] Meisel J. S. Hannigan G. D. Tyldsley A. S. SanMiguel A. J. Hodkinson B. P. Zheng Q. (2016). Skin microbiome surveys are strongly influenced by experimental design. J. investigative dermatology 136 (5), 947–956. 10.1016/j.jid.2016.01.016 PMC4842136 26829039

[B37] Mukherjee S. Mitra R. Maitra A. Gupta S. Kumaran S. Chakrabortty A. (2016). Sebum and hydration levels in specific regions of human face significantly predict the nature and diversity of facial skin microbiome. Sci. Rep. 6, 36062. 10.1038/srep36062 27786295 PMC5081537

[B38] NCBI (1996). Research on the menopause in the 1990s. Report of a WHO scientific group. World Health Organ. Tech. Rep. Ser. 866, 1–107. 8942292

[B39] Oh J. Byrd A. L. Park M. Kong H. H. Segre J. A. (2016). Temporal stability of the human skin microbiome. Cell 165 (4), 854–866. 10.1016/j.cell.2016.04.008 27153496 PMC4860256

[B40] Oh J. Conlan S. Polley E. C. Segre J. A. Kong H. H. (2012). Shifts in human skin and nares microbiota of healthy children and adults. Genome Med. 4 (10), 77. 10.1186/gm378 23050952 PMC3580446

[B41] Park M. G. Cho S. Oh M. M. (2023). Menopausal changes in the microbiome-A review focused on the genitourinary microbiome. Diagn. Basel, Switz. 13 (6), 1193. 10.3390/diagnostics13061193 PMC10047399 36980501

[B42] Patriarca M. T. Barbosa de Moraes A. R. Nader H. B. Petri V. Martins J. R. Gomes R. C. (2013). Hyaluronic acid concentration in postmenopausal facial skin after topical estradiol and genistein treatment: a double-blind, randomized clinical trial of efficacy. Menopause (New York, NY) 20 (3), 336–341. 10.1097/GME.0b013e318269898c 23435032

[B43] Pochi P. E. Strauss J. S. Downing D. T. (1979). Age-related changes in sebaceous gland activity. J. investigative dermatology 73 (1), 108–111. 10.1111/1523-1747.ep12532792 448169

[B44] Russo E. Di Gloria L. Cerboneschi M. Smeazzetto S. Baruzzi G. P. Romano F. (2023). Facial skin microbiome: aging-related changes and exploratory functional associations with host genetic factors, a pilot study. Biomedicines 11 (3), 684. 10.3390/biomedicines11030684 36979663 PMC10045008

[B45] Sator P. G. Schmidt J. B. Sator M. O. Huber J. C. Hönigsmann H. (2001). The influence of hormone replacement therapy on skin ageing: a pilot study. Maturitas 39 (1), 43–55. 10.1016/s0378-5122(00)00225-5 11451620

[B46] Sfriso R. Egert M. Gempeler M. Voegeli R. Campiche R. (2020). Revealing the secret life of skin - with the microbiome you never walk alone. Int. J. Cosmet. Sci. 42 (2), 116–126. 10.1111/ics.12594 31743445 PMC7155096

[B47] Shibagaki N. Suda W. Clavaud C. Bastien P. Takayasu L. Iioka E. (2017). Aging-related changes in the diversity of women's skin microbiomes associated with oral bacteria. Sci. Rep. 7 (1), 10567. 10.1038/s41598-017-10834-9 28874721 PMC5585242

[B48] Somboonna N. Wilantho A. Srisuttiyakorn C. Assawamakin A. Tongsima S. (2017). Bacterial communities on facial skin of teenage and elderly Thai females. Archives Microbiol. 199 (7), 1035–1042. 10.1007/s00203-017-1375-0 28391505

[B49] Sze M. Schloss P. (2019). The impact of DNA polymerase and number of rounds of amplification in PCR on 16S rRNA gene sequence data. mSphere 4, e00163–e00119. 10.1128/mSphere.00163-19 31118299 PMC6531881

[B50] Thody A. J. Shuster S. (1989). Control and function of sebaceous glands. Physiol. Rev. 69 (2), 383–416. 10.1152/physrev.1989.69.2.383 2648418

[B51] Thornton M. J. (2013). Estrogens and aging skin. Dermato-endocrinology. 5 (2), 264–270. 10.4161/derm.23872 24194966 PMC3772914

[B52] Townsend E. C. Kalan L. R. (2023). The dynamic balance of the skin microbiome across the lifespan. Biochem. Soc. Trans. 51 (1), 71–86. 10.1042/BST20220216 36606709 PMC9988004

[B53] Vieira A. T. Castelo P. M. Ribeiro D. A. Ferreira C. M. (2017). Influence of oral and gut microbiota in the health of menopausal women. Front. Microbiol. 8, 1884. 10.3389/fmicb.2017.01884 29033921 PMC5625026

[B54] Voegeli R. Campiche R. Biassin R. Rawlings A. V. Shackelford T. K. Fink B. (2023). Predictors of female age, health and attractiveness perception from skin feature analysis of digital portraits in five ethnic groups. Int. J. Cosmet. Sci. 45 (5), 672–687. 10.1111/ics.12877 37338195

[B55] von Graevenitz A. Bernard K. (2006). “The genus corynebacterium--medical,” in The prokaryotes: volume 3: archaea bacteria: firmicutes, actinomycetes. Editors Dworkin M. Falkow S. Rosenberg E. Schleifer K.-H. Stackebrandt E. (New York, NY: Springer New York), 819–842.

[B56] Zhou W. Fleming E. Legendre G. Roux L. Latreille J. Gendronneau G. (2023). Skin microbiome attributes associate with biophysical skin ageing. Exp. Dermatol. 32 (9), 1546–1556. 10.1111/exd.14863 37350224 PMC11128091

[B57] Zouboulis C. C. Blume-Peytavi U. Kosmadaki M. Roó E. Vexiau-Robert D. Kerob D. (2022). Skin, hair and beyond: the impact of menopause. Climacteric J. Int. Menopause Soc. 25 (5), 434–442. 10.1080/13697137.2022.2050206 35377827

