# Supplementary material for: Menopause and facial skin microbiomes: a pilot study revealing novel insights into their relationship
Source: Front Aging. 2024 Mar 21;5:1353082. doi: 10.3389/fragi.2024.1353082 (PMC10991793; doi:10.3389/fragi.2024.1353082)
Supplement: Supplementary file 3 [file Presentation1.pdf]

Supplemental Figure 1

A **B**

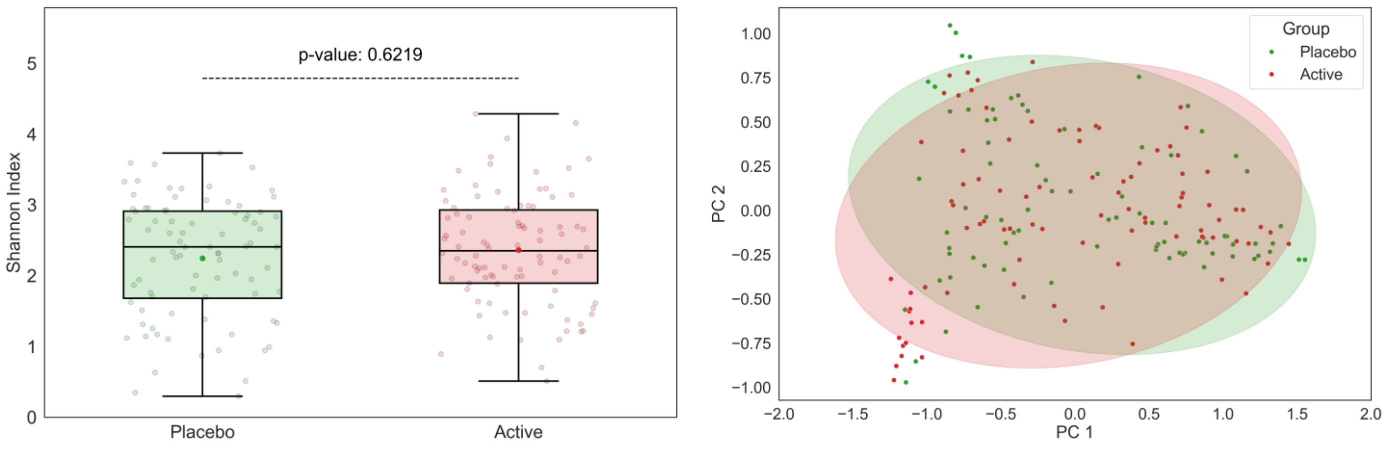

Supplemental Figure 1. Effect of topical formulations on facial skin microbiome profiles. A) One-week long topical application of either placebo or active formulations did not lead to different bacterial diversities, measured by Shannon's index, in samples from facial skin sites. Boxplot features are described in Figure 1. Respective p-values are indicated above the upper whiskers. B) Microbial compositions are similar between skin sites treated with either placebo or active formulations (PERMANOVA p=0.273, r^2=0.0136; PERMDISP p=0.845). For analyses shown here, all collected skin samples were used (n=176; equally distributed for the placebo and the verum group).

Supplemental Figure 2

A

B

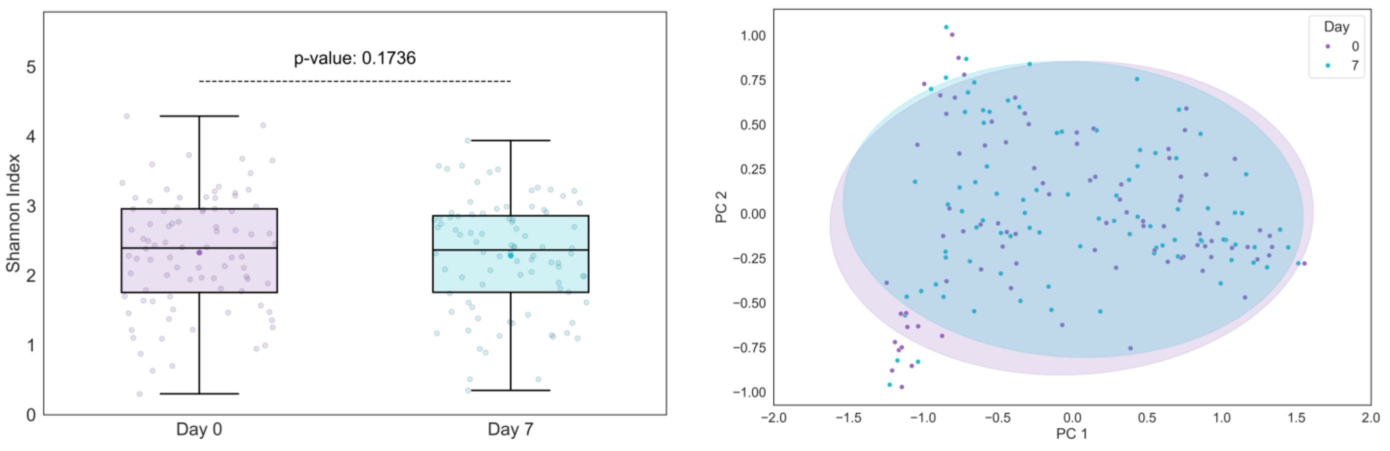

Supplemental Figure 2. Temporal stability of facial skin microbiome profiles. A) No significant differences in bacterial diversities, measured by Shannon's index, in samples from facial skin sites were detected over the course of one week. Boxplot features are described in Figure 1. Respective p-values are indicated above the upper whiskers. B) Skin microbial compositions are similar between the beginning and end of the one-week study (PERMANOVA p=0.816, r^2=0.00645; PERMDISP p=0.859). For analyses shown here, all collected skin samples were used (n=176; equally distributed for the different sampling times).

Supplemental Figure 3

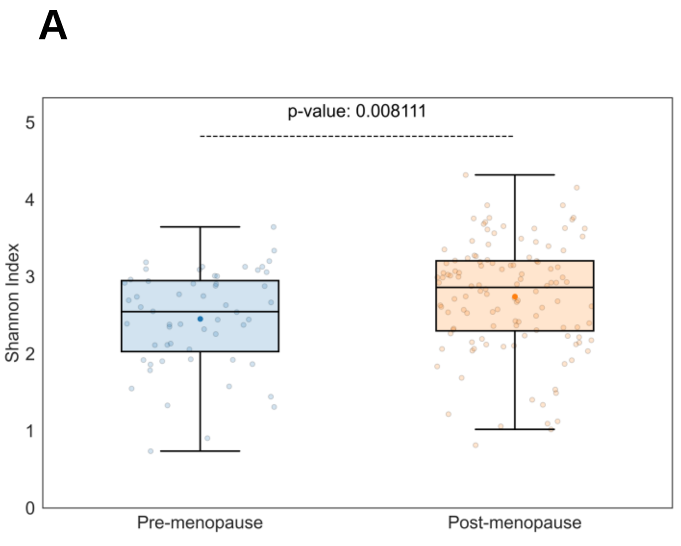

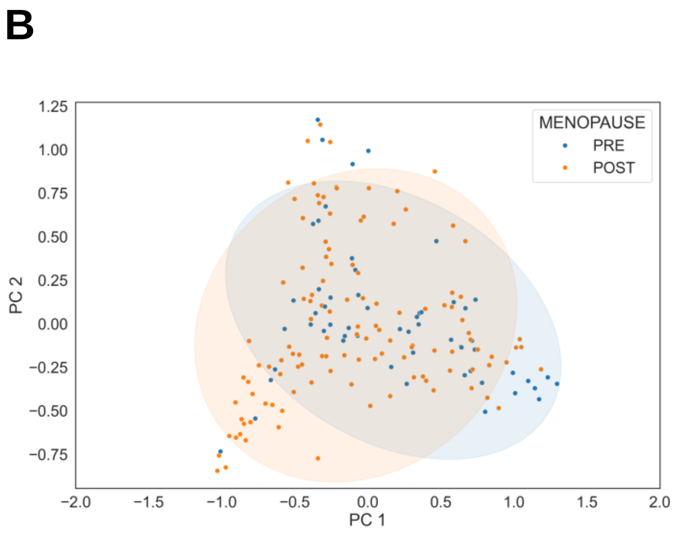

Supplemental Figure 3: Facial skin microbiome profiles after mathematical removal of the *Cutibacterium* genus. A) Genus-level bacterial diversity, measured by Shannon's index, is higher on postmenopausal than on premenopausal facial skin sites, even after removal of the *Cutibacterium* genus from the equation. Boxplot features are described in Figure 1. Respective p-values are indicated above the upper whiskers. B) Removal of the *Cutibacterium* genus maintains significant difference in skin microbial compositions between the two menopausal statuses (PERMANOVA p=0.00464, r^2=0.0284; PERMDISP p=0.809). Ellipses indicate 95% confidence intervals. For analyses shown here, all collected skin samples were used (n=176; 56 for the pre-, and 120 for the postmenopausal group).

Supplemental Figure 4

A

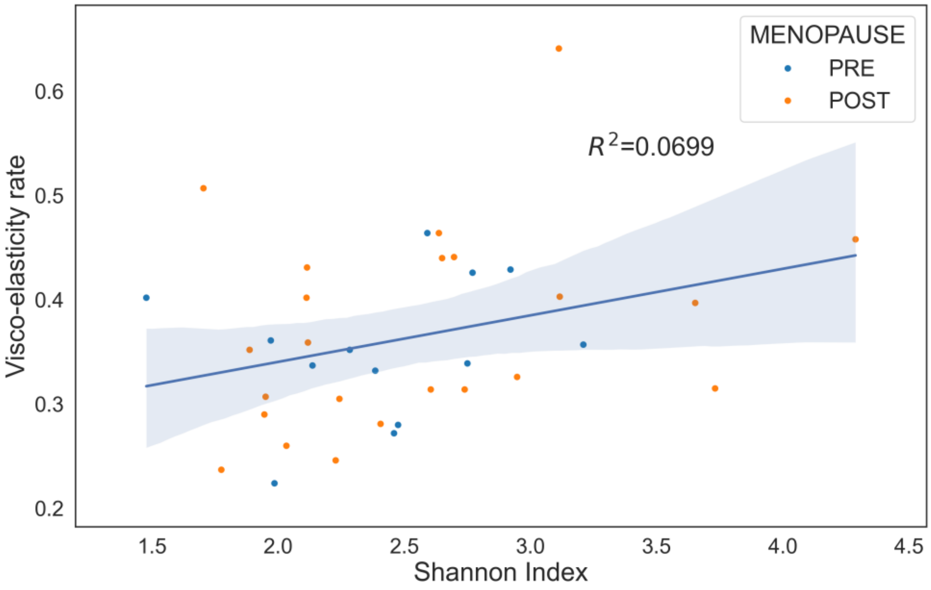

B

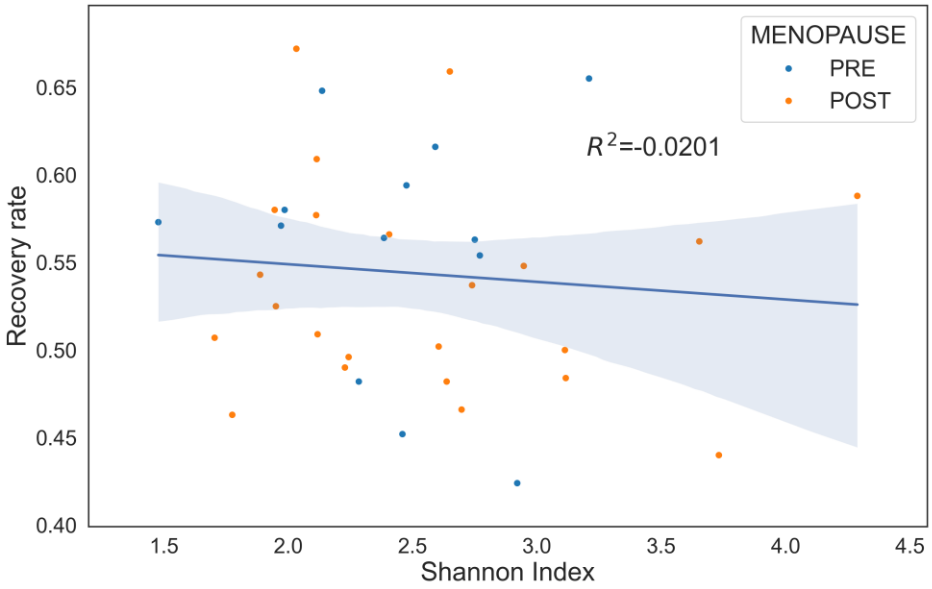

Supplemental Figure 4. Skin biophysical properties do not correlate with skin microbial diversity. Association between A) visco-elasticity rate, B) recovery rate and Shannon's index within pre- and postmenopausal groups (n=36; 13 for pre-, and 23 for postmenopausal subjects). Pearson correlation coefficient R^2 values are indicated. 95% confidence interval is shaded in blue.

Supplemental Figure 5

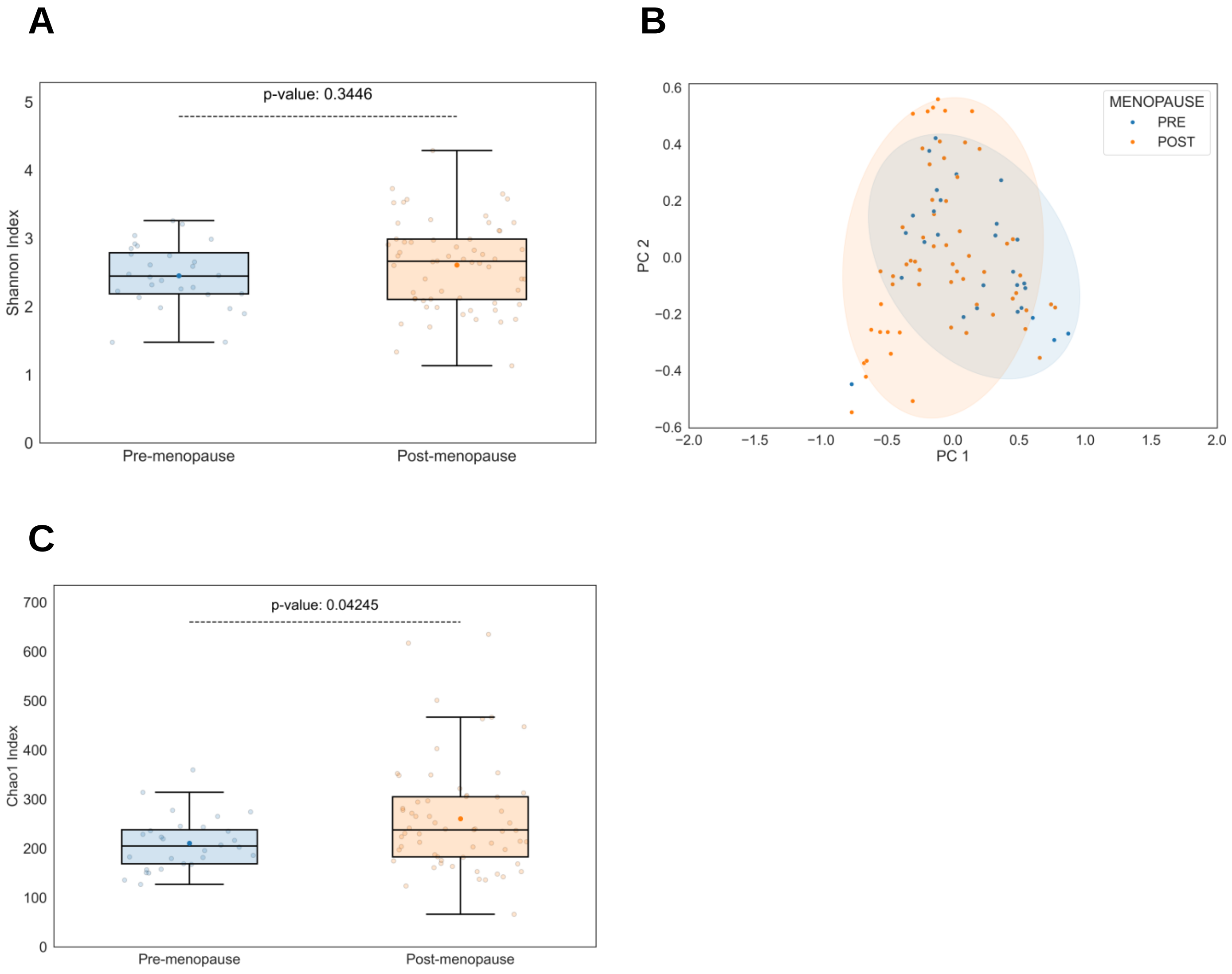

Supplemental Figure 5: Effect of menopausal status on cheek skin microbiome profiles. A) Bacterial diversity, measured by Shannon's index, is similar in samples from postmenopausal and premenopausal cheek skin sites. B) Cheek skin microbial composition is different between the two menopausal statuses (PERMANOVA p=0.019, r^2=0.0465; PERMDISP p=0.097). Ellipses indicate 95% confidence intervals. C) Bacterial richness, measured by Chao1 index, is significantly increased on postmenopausal cheek skin. Boxplot features are described in Figure 1. Respective p-values are indicated above the upper whiskers. For analyses shown here, all collected skin samples from cheek were used (n=88; 28 for pre-, and 60 for postmenopausal subjects).

Supplemental Figure 6

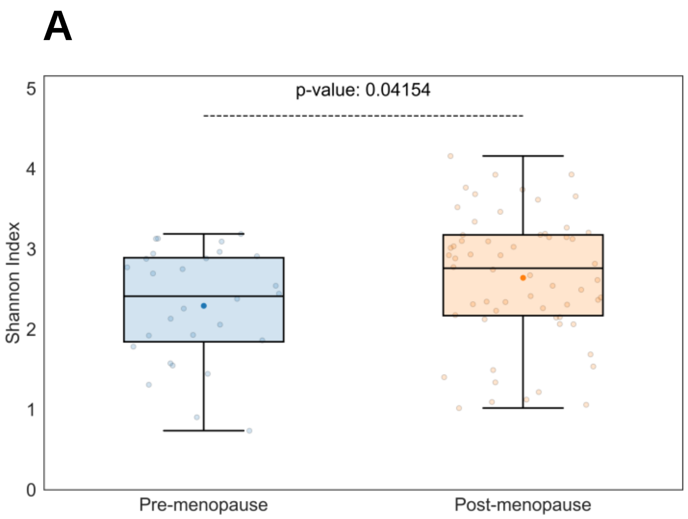

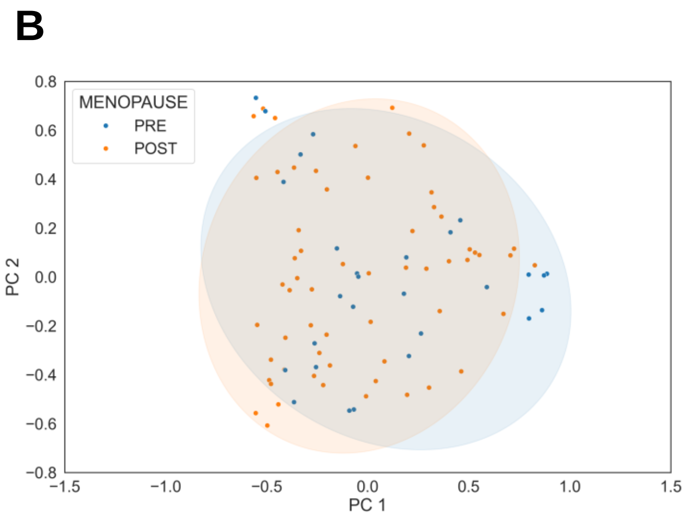

Supplemental Figure 6: Skin microbiome profiles on forehead after mathematical removal of *Cutibacterium* genus. A) Genus-level bacterial diversity, measured by Shannon's index, is higher on postmenopausal than on premenopausal forehead skin sites, even after removal of the *Cutibacterium* genus from the equation. Boxplot features are described in Figure 1. Respective p-values are indicated above the upper whiskers. B) Microbial compositions in forehead samples between the two menopausal statuses are similar in mathematical absence of the *Cutibacterium* genus (PERMANOVA p=0.191, r^2=0.03; PERMDISP p=0.598). Ellipses indicate 95% confidence intervals. For analyses shown here, all collected skin samples from forehead were used (n=88; 28 for pre-, and 60 for postmenopausal subjects).

Supplemental Figure 7

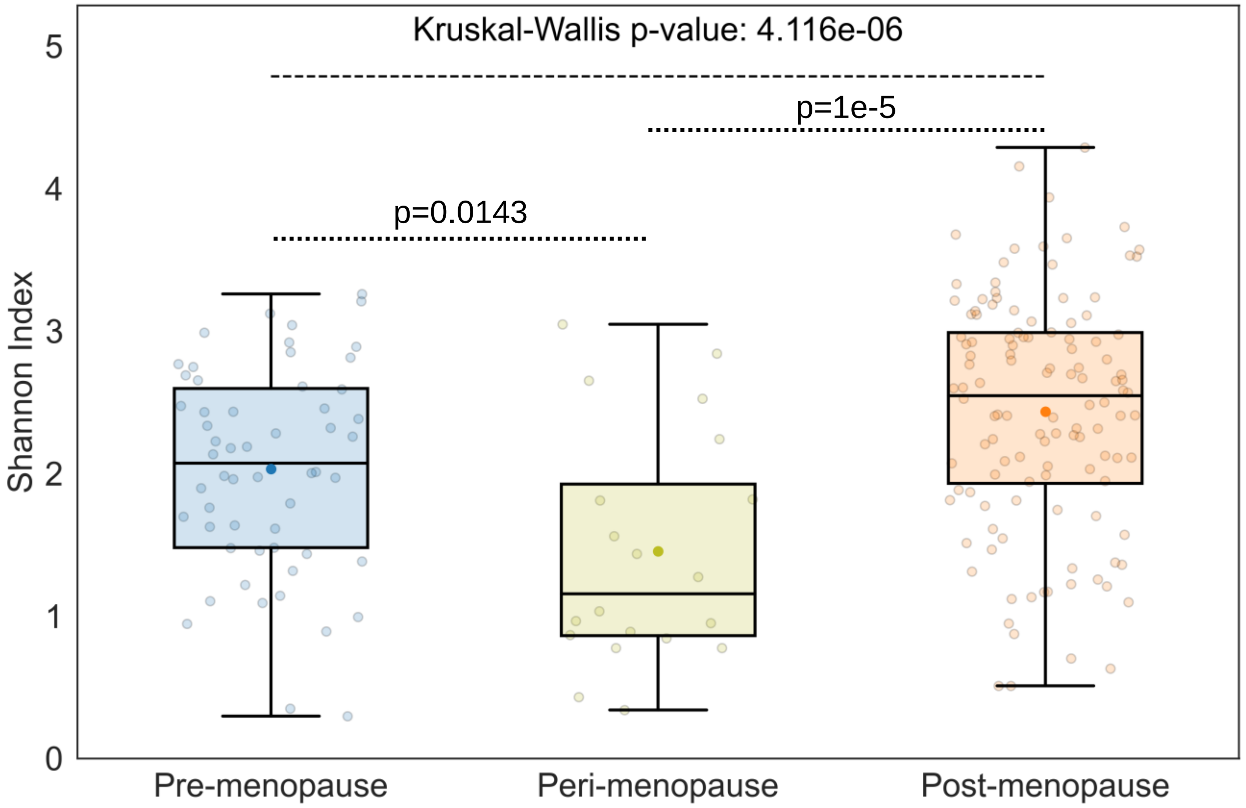

Supplemental Figure 7. Effect of menstrual cycle irregularity on facial skin microbial diversity. Genus-level bacterial diversity, measured by Shannon's index, is highest and lowest on post- and perimenopausal facial skin sites, respectively. Kruskal Wallis test was performed across the whole population. Between group differences were tested using post-hoc Conover. Boxplot features are described in Figure 1. Respective p-values are indicated above the upper whiskers. For analyses shown here, all collected skin samples were used (n=196; 56 for pre-, 20 for peri-, and 120 for postmenopausal subjects).
